# Alterations of Membrane Lipid Content Correlated With Chloroplast and Mitochondria Development in *Euglena gracilis*

**DOI:** 10.3389/fpls.2018.00370

**Published:** 2018-03-27

**Authors:** Shiori Shibata, Shin-ichi Arimura, Takahiro Ishikawa, Koichiro Awai

**Affiliations:** ^1^Graduate School of Integrated Science and Technology, Shizuoka University, Shizuoka, Japan; ^2^Graduate School of Agricultural and Life Sciences, The University of Tokyo, Tokyo, Japan; ^3^Precursory Research for Embryonic Science and Technology, Japan Science and Technology Agency, Saitama, Japan; ^4^Department of Life Science and Biotechnology, Faculty of Life and Environmental Science, Shimane University, Matsue, Japan; ^5^Core Research for Evolutional Science and Technology, Japan Science and Technology Agency, Tokyo, Japan; ^6^Research Institute of Electronics, Shizuoka University, Hamamatsu, Japan

**Keywords:** *Euglena gracilis*, membrane lipids, photosynthesis, respiration, thylakoid membranes, MitoTracker, confocal laser scanning microscopy

## Abstract

Euglenoids are unique protists that can grow photoautotrophically, photomixotrophically, and heterotrophically. Here we grew *Euglena gracilis* under these different growth conditions and determined cellular contents of seven membrane lipids and one storage lipid (triacylglycerol), which account for more than 94 mol% of total membrane lipids. We also describe the relationship among chloroplast and mitochondria developments with lipid contents, protein contents, and oxygen evolution/consumption rates. In photoautotrophic growth conditions, *E*. *gracilis* cells accumulated chlorophyll, photosynthetic proteins, and glycolipids typical to thylakoid membranes. The same occurred for the cells grown under photomixotrophic conditions with higher respiration rates. In heterotrophic conditions, *E*. *gracilis* cells had higher respiration rates compared to cells grown in other conditions with the accumulation of pyruvate: NADP+ oxidoreductase, a mitochondrial protein and phospholipid common in mitochondria. Cells were also observed using a confocal laser scanning microscope and found to show more chlorophyll autofluorescence when grown photoautotrophically and photomixotrophycally, and fluorescence of MitoTracker when grown photomixotrophically and heterotrophically. These results suggest that under illumination, *E*. *gracilis* develops functional thylakoid membranes with membrane lipids and proteins for photosynthesis. In the medium with glucose, the cells develop mitochondria with phospholipids and proteins for respiration. Possible application based on lipid analysis for the enhancement of wax ester or alkene synthesis is discussed.

## Introduction

Euglenoids are unicellular photosynthetic protists mostly found in freshwater, such as lakes, ponds, and rivers. Euglenoids are believed to acquire chloroplasts by secondary endosymbiosis with the alga that shares a common ancestor with current green algae (Gibbs, 1978; McFadden, 2001). Euglenoids are uniquely able to grow photoautotrophically, photomixotrophically, and heterotrophically. According to this feature, these organisms have been used for analysis of chloroplast development, especially in the model euglenoid *Euglena gracilis*. Since *E*. *gracilis* can grow heterotrophically, chloroplast development has been analyzed by illuminating dark-grown cells. When the *E*. *gracilis* cells transform from dark-heterotrophic to light-photoautotrophic growth, the cells start developing chloroplasts. *E*. *gracilis* cells are known to accumulate a storage carbohydrate, paramylon (β-1,3-glucose polymer) under heterotrophic growth conditions (Schwartzbach et al., 1975; Inui et al., 1982), and they utilize it during chloroplast development for synthesis of proteins, nucleic acids, and membrane lipids (Rosenberg and Pecker, 1964; Schiff and Schwartzbach, 1982; Osafune et al., 1990; Sumida et al., 2007). Light-grown cells have also been placed in the dark and analyzed for degradation of chloroplasts (Scheer and Parthier, 1982; Ferroni et al., 2009). These studies report the morphology of chloroplasts; content of photosynthetic proteins, pigments, and membrane lipids; and degradation of paramylon. However, there are few reports describing the relationship between membrane lipid composition and cell specialization in *E*. *gracilis*.

In *E*. *gracilis*, a wax ester, a type of storage lipid, is well analyzed for potential use as a biofuel (Inui et al., 1982, 1984, 2017). This wax ester is mainly composed of C14:0 saturated fatty acid, myristic acid, and myristyl alcohol (Hulanicka et al., 1964). It is known to accumulate especially under hypoxic conditions by consuming the paramylon to obtain energy without respiration (Inui et al., 1982). Both wax ester and membrane lipids are made from fatty acids, there are not much information on membrane lipids of *E*. *gracilis*. Early reports described the lipid metabolism (Hulanicka et al., 1964) and the effect of light intensity on lipid composition (Constantopoulos and Bloch, 1967), but these reports mainly analyzed fatty acid composition and only described membrane lipids as the sums of polar lipids or phospholipids. Some reports describe the membrane lipid composition of *E*. *gracilis*, especially thylakoid glycolipids monogalactosyldiacylglycerol (MGDG), digalactosyldiacylglycerol (DGDG) and sulfoquinovosyldiacylglycerol (SQDG) (Rosenberg, 1963; Rosenberg and Pecker, 1964; Rosenberg et al., 1966). More recently, comprehensive analyses of membrane lipids were done with classical (Regnault et al., 1995) or more advanced techniques (LC-MS/MS) (Ogawa et al., 2014). The former one reports only five membrane lipids including MGDG, DGDG, phosphatidylglycerol (PG), phosphatidylcholine (PC), and phosphatidylethanolamine (PE). The latter one showed unusual lipid contents (lacking DGDG and SQDG with a very high content of sphingomyelin). Phosophatidylinositol (PI) is also described as a minor component in some reports (Calvayrac and Douce, 1970; Fujita et al., 1995). Therefore, more detailed analyses of lipid compositions of *E*. *gracilis* are needed.

In this article, we present comprehensive analysis of lipids in *E*. *gracilis* by a combination of traditional TLC-based methods with LC-MS/MS. Based on this, we analyzed the relationship between membrane lipid contents and chloroplast/mitochondria developments with oxygen evolution/consumption rates, quantum efficiency of photosystem II (PSII), amounts of chloroplastic/mitochondrial proteins, and a confocal laser scanning microscopy.

## Materials and Methods

### Growth Conditions

*Euglena gracilis* Z was cultured in 200-ml flasks containing 100 ml of Cramer–Myers (CM) medium (Cramer and Myers, 1952) for photoautotrophic growth at 26°C under continuous light (100 μmol⋅photons m^-2^⋅s^-1^) with rotary shaking at 120 rpm. For photomixotrophic growth, CM medium with 0.6% (w/v) glucose (CM+Glc) was used under the same conditions. For the heterotrophic growth, cells were cultured in CM+Glc medium in the same condition, but the flasks were completely wrapped with aluminum foil. To obtain growth curves, algal cultures were diluted with fresh medium at an initial cell number of 3.0 × 10^3^, and the cell number was counted using the Cellometer (Auto T4, Nexcelom, United States) every 24 h.

### Chlorophyll Contents, Oxygen Evolution Rates, and Chlorophyll Fluorescence

Chlorophyll content was measured as described (Arnon, 1949). The oxygen evolution rate of intact cells was measured with a Clark-type oxygen electrode (Hansatech Instruments Ltd.) and a LED lamp (CCS Inc., Kyoto, Japan). Chlorophyll fluorescence measurements were performed with a Dual-PAM system (Heinz Walz GmbH). PSII quantum efficiency was measured as (Fm-Fo)/Fm, where Fm is the maximum PSII fluorescence obtained with a red saturating pulse (635 nm, 300 ms duration, 20,000 μmol photons m^-2^ s^-1^) and Fo is the minimum fluorescence obtained after 10 min of far red light (intensity setting 20) to ensure a state 1 transition.

### Protein Extraction, SDS-PAGE, and Western Blot Analysis

For protein extraction, 2 ml of culture (∼1 × 10^6^ cells) was centrifuged at 16,000 × *g*, and the precipitated cells were frozen with liquid nitrogen. These cells were lysed five times by a homogenizer (Micro Smash MS-100R, TOMY) at 2,000 rpm for 20 s. Then the powdered cells were suspended in 200 μl of the resuspend buffer [50 mM HEPES (pH 7.0), 25 mM CaCl_2_, 5 mM MgCl_2_, 10% (v/v) glycerol, 1 mM PMSF, and 5 mM 6-aminocaproic acid] (Barthel et al., 2013) and used for Western blot analysis. For protein content analysis, aliquots of the suspended cells were mixed with the same volume of detergent solution [60 mM Tris-HCl (pH6.8), 2% SDS (w/v)]. A BCA Protein Assay Kit (Thermo Fischer Scientific) was used with BSA as a standard.

Laemmli SDS-PAGE (Laemmli, 1970) was performed using polyacrylamide gels containing 10% (w/v) acrylamide. Proteins from the same number of cells (∼5 × 10^4^ cells) were applied to each lane. Samples were mixed with loading buffer with 175 mM DTT and not heat-denatured to avoid aggregation of membrane proteins. The gels were stained with Coomassie Brilliant Blue R-250 (CBB). Pre-stained Protein Markers (Broad Range) for SDS-PAGE (Nacalai Tesque) were used to calibrate the gels.

For immunodetection, rabbit antibodies against PsbC (AS111787, CP43), PsbO (AS06-142-33), and RbcL (AS03037) (Agrisera) were used at a 1:3,000 dilution. Polyclonal antibodies against spinach PsbA/D (a gift from Dr. Masahiko Ikeuchi at The University of Tokyo) and Euglena pyruvate: NADP+ oxidoreductase (PNO, a gift from Dr. Masami Nakazawa at Osaka Prefecture University) were used at the same dilution. These antibodies were detected with an anti-rabbit horseradish peroxidase-coupled antibody (ab97051, Abcam) at a dilution of 1:10,000 using Can Get Signal Immunoreaction Enhancer Solution (Toyobo) followed by development with Western Lightning Plus-ECL (Perkin Elmer).

### Fluorescence Microscopy

Fluorescence microscopy was carried out as described (Nagaoka et al., 2017). Briefly, Euglena cells were stained with MitoTracker Orange CMTMRos (Life Technologies) for 30 min and washed five times with CM medium before observations by a fluorescent microscope (Nikon, ECLIPSE Ti) equipped with a confocal laser scanning microscope system (Nikon, C1Si).

### Lipid Analysis

Lipids were extracted as described (Bligh and Dyer, 1959) and separated by TLC. For membrane lipid analysis, a solvent system of chloroform: methanol: petroleum ether: acetone: acetic acid: H_2_O (20: 15: 10: 5: 1.3: 1, v/v) (Suzuki et al., 2016) was used for initial separation. Then, detected spots were further separated by the solvent systems hexane: tetrahydrofuran: 2-propanol: H_2_O (50: 0.5: 35: 3, v/v) (Hölzl et al., 2005), chloroform: methanol: acetic acid: H_2_O (34: 5: 5: 0.8, v/v) (Nichols and James, 1964), or chloroform: methanol: H_2_O (65: 25: 4, v/v) (Allen et al., 1966). For neutral lipid analysis, a solvent system of hexane: diethyl ether: acetic acid (80: 20: 1 or 90: 15: 1, v/v) (Kalscheuer and Steinbuchel, 2003) was used.

Separated lipids were determined by TLC co-chromato-graphed with standard lipids and liquid chromatography-mass spectrometry (LC-MS, Shimadzu LCMS-2010A system) as described (Okazaki et al., 2013).

For quantitative and qualitative analysis of fatty acids attached to lipids, each separated spot was scraped off and fatty acid methyl esters (FAMEs) were prepared. FAMEs were then analyzed by gas chromatography (GC) equipped with a flame ionization detector (FID) for quantitative analysis, and GC-mass spectrometry (MS) for qualitative analysis. GC-FID was carried out as described (Awai et al., 2014) with slight modification. Shimadzu GC-2014 equipped with a FID on a capillary column (BPX90, 60 m × 0.25 mm, SGE Analytical Science) was used. The column temperature was gradually increased from 140°C to 240°C at a rate of 5°C/min. The injector and detector temperature were both 250°C. The linear velocity of carrier gas (He) was 25 cm/min. GC-MS was carried out using a Shimadzu GCMS-QP2010SE under the same condition as GC-FID. The ion source temperature was set to 200°C and both the injection port and interface temperature were 250°C.

## Results

### Growth and Photosynthetic Activity of *E*. *gracilis* Under Different Growth Conditions

**FIGURE 1:**
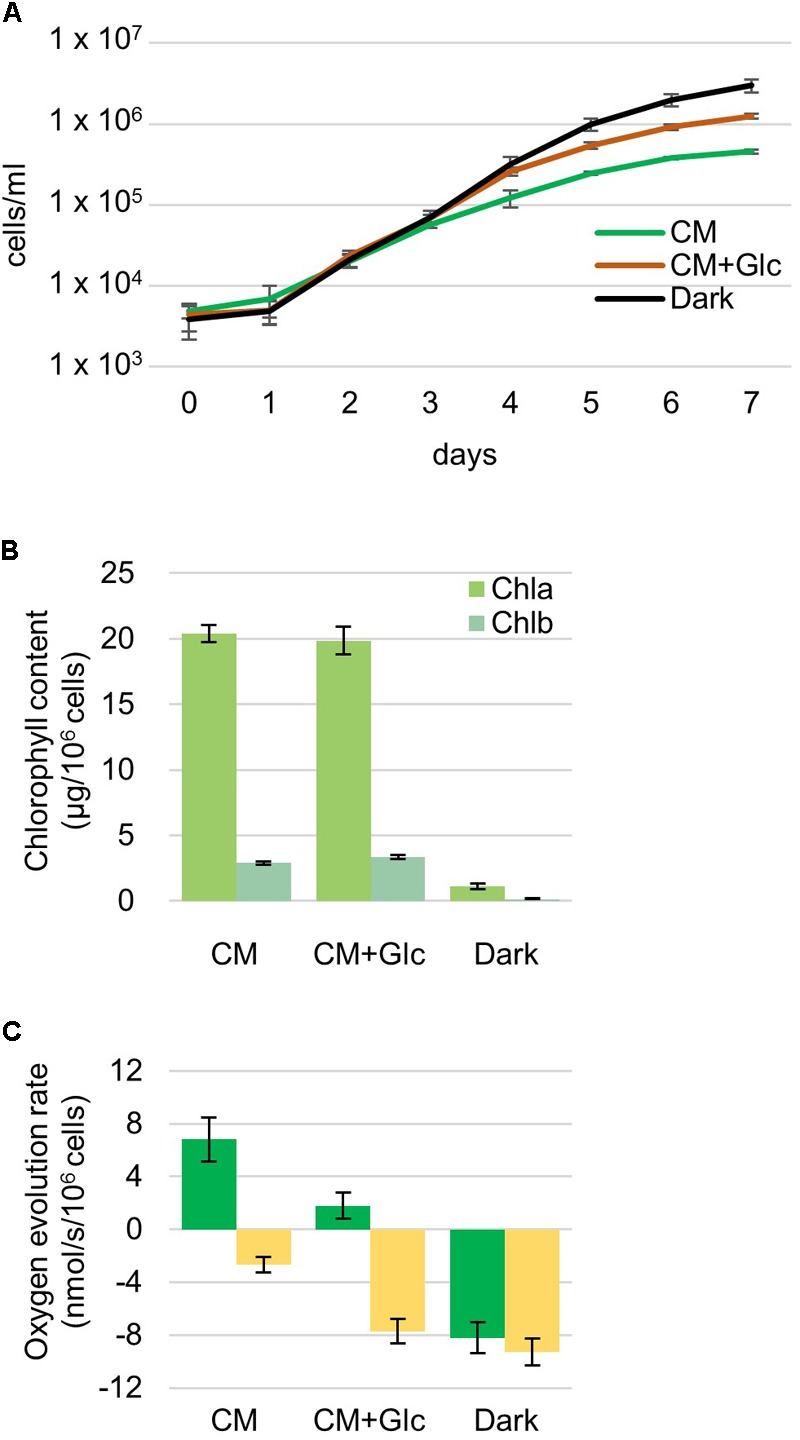
Properties of Euglena cells grown under photoautotrophic, photomixotrophic and heterotrophic conditions. **(A)** Growth curve of the cells. Algal cultures were diluted with fresh medium at an initial cell number approximately 4,000 and grown for 7 days. Error bars indicate the SE based on six independent experiments. **(B)** Chlorophyll contents per 10^6^ cells. Error bars indicate the SE based on at least twelve independent experiments. **(C)** Oxygen evolution and consumption rates. Oxygen evolution rates were measured under the growth light intensity (105 μmol photons m^-2^⋅s^-1^). Green bar: oxygen evolution rate, yellow bar: oxygen consumption rate. Error bars indicate the SE based on at least three independent experiments.

*Euglena gracilis* cells were grown under different growth conditions, namely photoautotrophic (CM: CM medium, 100 μmol photons m^-2^ s^-1^), photomixotrophic (CM+Glc: CM medium with 0.6% glucose, 100 μmol photons m^-2^ s^-1^) and heterotrophic (Dark: CM medium with 0.6% glucose, dark) conditions. First, growth rates of *E*. *gracilis* cells in each condition were compared. As shown in **Figure 1A**, the cells grew at similar rates under all conditions and the Dark condition showed the highest cell number compared with the others. Chlorophyll content was almost the same among cells grown in CM and CM+Glc media [chlorophyll (Chl) *a* + *b* per 10^6^ cells ± SE, *n* ≥ 12: CM, 23.30 ± 0.75 μg Chl; CM+Glc, 23.22 ± 1.20 μg Chl] (**Figure 1B**). On the other hand, in Dark conditions, chlorophyll content was much lower than in the cells illuminated by light (1.25 ± 0.28 μg Chl). We next analyzed oxygen evolution rate in the cells. As expected from the lower Chl contents, oxygen consumption was observed in the cells cultured in Dark conditions even under light (**Figure 1C**). This consumption rate was slightly lower than the oxygen consumption rate measured under dark conditions (respiration), indicating that the dark-grown cells still have a weak photosynthetic activity. Cells grown under CM conditions showed a higher oxygen evolution rate compared with the cells grown under CM+Glc conditions. This can be explained by a higher rate of oxygen consumption in the cells of CM+Glc conditions (**Figure 1C**). In fact, the total amount of oxygen evolution/consumption was almost the same in the cells grown in CM and CM+Glc conditions (CM, 9.48 nmol/s/10^6^ cells; CM+Glc, 9.53 nmol/s/10^6^ cells). These results suggest that cells grown under light have similar photosynthetic activities, but addition of glucose stimulated respiration activity in the cells grown in CM+Glc conditions. The protein content of the cells was also analyzed (protein per 10^6^ cells ± SE, *n* = 3: CM, 269.63 ± 17.87 μg Protein; CM+Glc, 253.06 ± 9.93 μg Protein; Dark, 200.61 ± 11.13 μg Protein). Dark-grown cells had less proteins than others but still to the same extent. Compositional changes in the protein profile were shown using a CBB-stained acrylamide gel (**Supplementary Figure S1**).

### Chloroplast Development in Illuminated *E. gracilis* Cells

**FIGURE 2:**
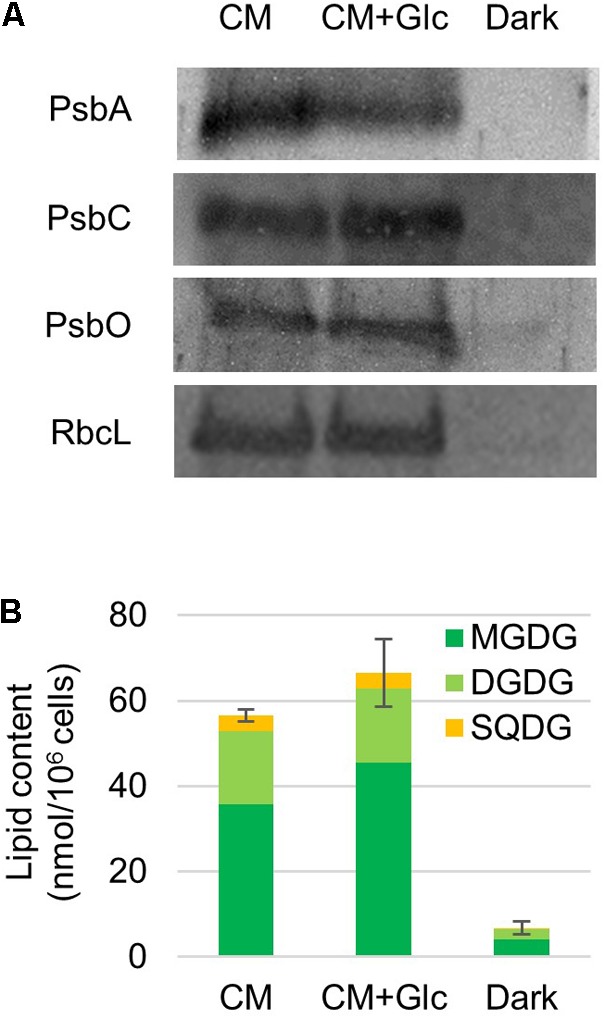
Photosynthetic protein and thylakoid glycolipid levels in cells grown under photoautotrophic, photomixotrophic and heterotrophic conditions. **(A)** Western blot analysis of photosynthetic proteins. Antibodies used are indicated on the left. **(B)** Thylakoid glycolipid content. Error bar shows SE (*n* = 3) of the total amount of glycolipids.

It was expected from the above-mentioned results that chloroplasts are developed under light regardless of the carbon source (CO_2_ or CO_2_ + Glc). To confirm this finding, we analyzed protein levels of photosynthetic complexes and thylakoid lipids. First, photosynthetic proteins, namely proteins from PSII [PsbA/D (D1/D2), PsbC (CP43) and PsbO] and the Rubisco large subunit were analyzed. As shown in **Figure 2**, these proteins were abundant in the cells grown under light (CM and CM+Glc conditions). On the other hand, these proteins were less abundant in the cells grown under Dark conditions.

We then analyzed the amount of thylakoid lipids in the cells grown under each condition. The major lipids in the thylakoid membranes are MGDG, DGDG, SQDG, and PG (Kobayashi et al., 2016). However, PG is also found in other membrane system such as the ER and mitochondria. We analyzed the amount of the glycolipids, namely MGDG, DGDG, and SQDG. Galactolipids MGDG and DGDG accumulated to high levels in the cells grown in both CM and CM+Glc conditions. Together with SQDG, the total amounts of glycolipids were 56.58 ± 1.36 nmol/10^6^ cells in CM conditions and 66.55 ± 7.89 nmol/10^6^ cells in CM+Glc conditions. In contrast, in Dark conditions, the amount of glycolipids was about ten times lower (6.80 ± 1.48 nmol/10^6^ cells), indicating that the thylakoid membrane was not developed well in dark-grown cells. We also analyzed the quantum efficiency of PSII, yet this could not be detected in the cells grown in Dark conditions. This is probably because there are not enough PSII proteins, such as PsbA/D, PsbC, and PsbO (**Figure 2A**). Conversely, the cells grown under light showed similar efficiencies regardless of carbon source (CM condition, 0.66 ± 0.01; CM+Glc conditions, 0.62 ± 0.02). These results suggest that *E. gracilis* grown under light accumulated proteins of photosynthetic complexes and thylakoid glycolipids to construct active thylakoid membranes.

### Mitochondria Development in *E. gracilis* Under Heterotrophic Conditions

**FIGURE 3:**
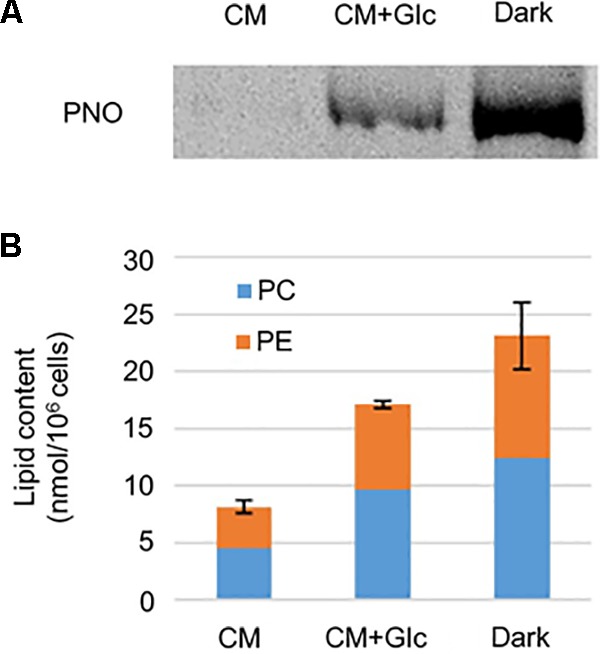
Mitochondrial protein and phospholipid levels in cells grown under photoautotrophic, photomixotrophic and heterotrophic conditions. **(A)** Western blot analysis of a mitochondrion protein, PNO. **(B)** Phospholipid content. Error bar shows SE (*n* = 3) of total amount of two extraplastidic phospholipids, PC and PE.

As shown in **Figure 2**, respiration rate increased under heterotrophic conditions. To monitor whether the *E*. *gracilis* cells developed mitochondria in those conditions, we analyzed mitochondrial protein content. We used an antibody against pyruvate: NADP+ oxidoreductase (PNO) which is involved in the formation of acetyl-CoA and localizes in mitochondria (Nakazawa et al., 2017). As expected, under heterotrophic conditions, *E*. *gracilis* cells accumulated PNO compared to the cells grown under CM conditions (**Figure 3A**). We also detected PNO in the cells grown under CM+Glc conditions, but it was less than that of the Dark conditions.

We next analyzed content of phospholipids, PC, and PE, which are typical membrane lipids in mitochondria. As shown in **Figure 3B**, the amounts of these lipids were highest in the cells grown under Dark conditions (23.12 ± 2.93 nmol/10^6^ cells). The cells grown under CM+Glc conditions had fewer lipids (17.08 ± 0.36 nmol/10^6^ cells) than the cells grown in the dark, but still more than twice as much as the cells grown under CM conditions (8.12 ± 0.56 nmol/10^6^ cells). Together with the respiration rate and amount of PNO, these results suggest that mitochondria are well developed when a carbon source is added to the medium.

**FIGURE 4:**
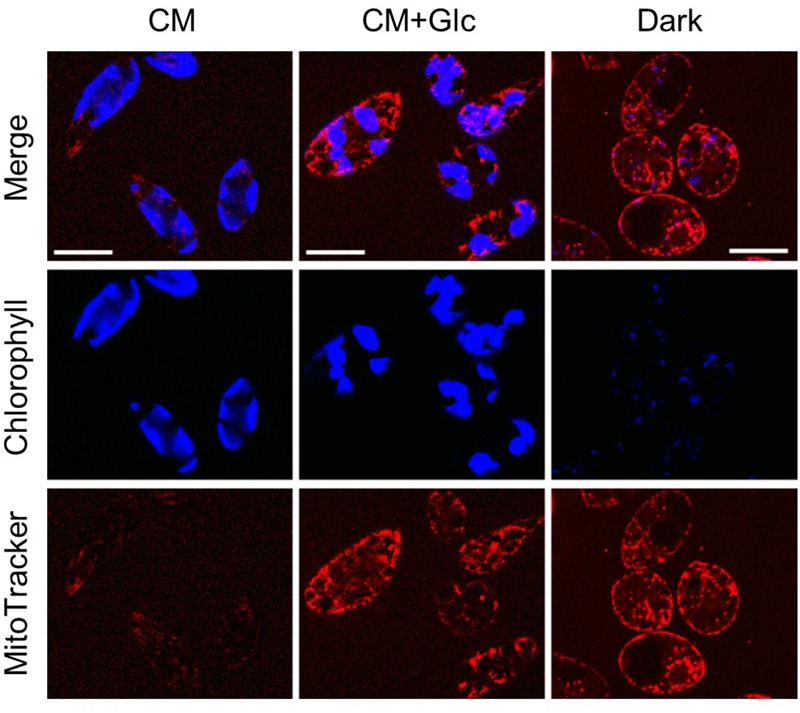
Confocal laser scan microscopy of the cells grown under photoautotrophic, photomixotrophic, and heterotrophic conditions. Typical images of the cells grown in each culture were shown. Bar = 20 μm.

To confirm these phenomena, we observed the cells grown under each condition using a confocal laser scanning microscope. As shown in **Figure 4**, auto-fluorescence of chlorophyll (indicated by blue) was seen clearly in the cells grown under light (CM and CM+Glc conditions). The cells grown in Dark conditions showed faint signal of chlorophyll, correlate with the chlorophyll content of the cells (**Figure 1B**). Mitochondria were stained using MitoTracker and indicated by pink. The cells grown with glucose (CM+Glc and Dark conditions) showed strong signals of MitoTracker compared with the cells grown under CM conditions, which also correlate with the respiration activity; contents of PNO and phospholipids. These results agree with the idea that lipid contents correlated with chloroplasts and mitochondria development.

### Revisiting Lipid and Fatty Acid Contents of *E. gracilis*

Lipid content of *E*. *gracilis* is summarized in **Table 1**. We detected seven membrane lipids as major constituents, including three glycolipids (MGDG, DGDG, and SQDG) and four phospholipids (PC, PE, PG, and PI). This composition is very similar to the lipid content of other eukaryotic phototrophs, such as the model dicot *Arabidopsis thaliana* (Browse et al., 1986). According to the fatty acid base calculation, we succeeded in detecting more than 94 mol% of membrane lipids in *E*. *gracilis*. The other 6 mol% of membrane lipids (others in **Table 1**) could not be determined because they did not co-migrate with the available standards, such as sphingomyelin or phosphatidylserine and were difficult to analyze by LC-MS/MS because of their low abundancies.

**Table 1:** Glycerolipid content of *E*. *gracilis* cells grown under photoautotrophic, photomixotrophic and heterotrophic conditions.

	CM	CM+Glc	Dark
MGDG	35.73 ± 2.11	45.46 ± 6.93	4.11 ± 0.99
DGDG	17.22 ± 0.69	17.38 ± 1.27	2.33 ± 0.41
SQDG	3.63 ± 0.11	3.71 ± 0.42	0.35 ± 0.09
PG	2.94 ± 0.10	2.67 ± 0.08	0.47 ± 0.15
PC	4.54 ± 0.34	9.64 ± 0.52	12.40 ± 0.80
PE	3.58 ± 0.32	7.44 ± 0.39	10.71 ± 2.14
PI	0.37 ± 0.04	0.61 ± 0.07	0.40 ± 0.08
others	2.83 ± 0.33	3.25 ± 0.23	1.95 ± 0.29
TAG	0.39 ± 0.06	1.58 ± 0.65	2.85 ± 0.53

*Values are nmol/10^*6*^ cells ± SE (*n* = 3).*

Fatty acid contents of each membrane lipid in the different growth conditions are described in **Tables 2–4**. The fatty acid content was similar regardless of growth conditions, except for PG. PG in the cells grown under Dark condition had much more γ-linolenic acid [18:3(n-6)] compared to the cells grown under CM or CM+Glc conditions [more α-linolenic acid: 18:3(n-3)]. Thylakoid galactolipids, namely MGDG and DGDG, were found to have more hexadeca-4,7,10,13-tetraenoic acid (16:4). Phospholipids such as PC and PE contained more of the long chain fatty acid, eicosatetraenoic acid (20:4).

**Table 2:** Fatty acid composition of membrane lipids from *E*. *gracilis* cultured under CM conditions.

	14:0	15:0	16:0	16:1	16:2	16:3	16:4	17:3	18:0	18:1(9)	18:1(11)	18:2	18:3 (*n*-6)	18:3 (*n*-3)	18:4	20:2	20:3 (*n*-6)	20:4	20:5	22:2
MGDG	0.18 ± 0.03	0.09 ± 0.03	0.35 ± 0.07	2.22 ± 0.34	15.46 ± 3.58	1.32 ± 0.29	24.09 ± 6.28	8.08 ± 2.09	0.04 ± 0.03	0.15 ± 0.03	0.26 ± 0.01	11.66 ± 3.33	0.16 ± 0.04	30.73 ± 8.22	ND	0.02 ± 0.01	0.42 ± 0.03	0.74 ± 0.08	1.39 ± 0.10	2.65 ± 0.17
DGDG	0.26 ± 0.02	0.06 ± 0.06	5.13 ± 0.35	3.52 ± 0.43	23.83 ± 0.38	0.29 ± 0.01	7.48 ± 0.48	12.61 ± 0.19	0.85 ± 0.39	0.17 ± 0.00	2.17 ± 0.49	18.57 ± 0.45	0.20 ± 0.02	22.06 ± 0.92	0.01 ± 0.01	0.04 ± 0.02	0.30 ± 0.04	0.47 ± 0.05	0.51 ± 0.03	1.45 ± 0.21
SQDG	2.90 ± 0.34	ND	59.38 ± 1.57	2.41 ± 0.28	2.88 ± 0.11	ND	0.66 ± 0.11	1.16 ± 0.10	1.60 ± 0.62	ND	2.94 ± 0.12	9.69 ± 0.39	0.20 ± 0.13	14.53 ± 1.30	ND	ND	0.18 ± 0.09	0.16 ± 0.08	0.28 ± 0.14	1.02 ± 0.08
PG	1.02 ± 0.34	ND	4.60 ± 0.71	50.09 ± 2.58	1.28 ± 0.21	ND	0.85 ± 0.22	0.13 ± 0.13	1.72 ± 1.09	ND	2.30 ± 0.09	26.98 ± 0.15	0.77 ± 0.08	10.04 ± 0.72	ND	ND	0.06 ± 0.06	ND	ND	0.16 ± 0.09
PC	9.84 ± 0.62	0.41 ± 0.27	17.95 ± 0.90	0.41 ± 0.13	0.06 ± 0.06	ND	ND	ND	1.32 ± 0.30	ND	8.75 ± 0.35	1.05 ± 0.31	0.15 ± 0.08	0.38 ± 0.38	ND	4.91 ± 0.13	5.31 ± 0.18	20.42 ± 0.38	17.73 ± 0.43	11.3 ± 0.53
PE	4.31 ± 0.32	0.32 ± 0.32	26.16 ± 1.47	3.23 ± 0.15	1.02 ± 0.18	ND	0.17 ± 0.17	0.20 ± 0.11	1.05 ± 0.19	ND	34.25 ± 0.63	3.34 ± 0.37	ND	1.02 ± 0.32	0.06 ± 0.06	1.82 ± 0.23	0.86 ± 0.04	17.09 ± 0.52	4.05 ± 0.14	1.06 ± 0.05
PI	8.32 ± 2.07	1.39 ± 1.39	57.51 ± 1.57	ND	ND	ND	ND	ND	4.12 ± 2.08	ND	ND	ND	ND	0.70 ± 0.70	ND	12.39 ± 2.66	ND	12.72 ± 2.51	2.87 ± 1.53	ND
others	12.54 ± 2.39	0.70 ± 0.36	47.62 ± 0.31	2.31 ± 0.51	1.39 ± 0.34	ND	ND	0.18 ± 0.18	11.84 ± 5.51	ND	6.64 ± 0.98	3.12 ± 0.33	ND	0.86 ± 0.17	ND	1.53 ± 0.76	ND	2.71 ± 0.70	8.40 ± 0.99	0.16 ± 0.16

*Each value is represented as mol%. SE is based on three independent experiments. ND, not detected (<0.01 mol%).*

**Table 3:** Fatty acid composition of membrane lipids from *E*. *gracilis* cultured under CM+Glc conditions.

	14:0	15:0	16:0	16:1	16:2	16:3	16:4	17:3	18:0	18:1(9)	18:1(11)	18:2	18:3 (*n*-6)	18:3 (*n*-3)	18:4	20:2	20:3 (*n*-6)	20:4	20:5	22:2
MGDG	0.26 ± 0.05	0.10 ± 0.01	0.71 ± 0.04	2.28 ± 0.24	13.64 ± 2.00	0.59 ± 0.02	22.42 ± 1.81	11.61 ± 0.63	0.09 ± 0.02	0.19 ± 0.02	0.96 ± 0.27	11.91 ± 1.47	0.26 ± 0.04	33.87 ± 1.94	ND	0.03 ± 0.01	0.10 ± 0.03	0.29 ± 0.05	0.41 ± 0.09	0.26 ± 0.08
DGDG	0.30 ± 0.05	0.12 ± 0.05	6.17 ± 0.43	3.02 ± 0.58	19.51 ± 2.67	0.06 ± 0.02	3.89 ± 0.41	19.55 ± 2.13	0.10 ± 0.02	0.23 ± 0.03	4.56 ± 0.97	15.11 ± 2.80	0.30 ± 0.06	26.51 ± 4.20	ND	0.02 ± 0.01	0.07 ± 0.02	0.19 ± 0.02	0.13 ± 0.02	0.17 ± 0.04
SQDG	2.42 ± 0.24	0.43 ± 0.18	55.65 ± 1.20	1.93 ± 0.37	2.68 ± 0.48	ND	0.29 ± 0.11	1.77 ± 0.35	1.59 ± 0.07	ND	6.95 ± 0.56	9.92 ± 1.28	0.16 ± 0.11	15.87 ± 2.13	0.03 ± 0.03	ND	0.03 ± 0.03	0.03 ± 0.03	0.05 ± 0.05	0.17 ± 0.06
PG	1.12 ± 0.29	0.05 ± 0.05	5.66 ± 1.40	40.26 ± 4.58	4.41 ± 0.93	0.14 ± 0.14	8.18 ± 4.71	3.40 ± 1.77	0.41 ± 0.15	0.06 ± 0.06	2.77 ± 0.43	20.34 ± 3.08	1.66 ± 0.83	11.17 ± 0.55	ND	ND	0.12 ± 0.11	0.06 ± 0.06	0.07 ± 0.04	0.11 ± 0.11
PC	9.75 ± 1.74	1.56 ± 0.42	16.37 ± 1.86	1.20 ± 0.17	0.09 ± 0.06	0.02 ± 0.02	0.08 ± 0.03	ND	1.14 ± 0.41	0.11 ± 0.11	7.40 ± 0.43	1.88 ± 0.13	0.47 ± 0.06	0.89 ± 0.34	0.11 ± 0.04	6.02 ± 1.11	11.31 ± 3.40	18.38 ± 1.03	14.29 ± 1.49	8.93 ± 2.95
PE	5.35 ± 0.71	1.96 ± 0.88	23.09 ± 1.52	6.87 ± 0.32	3.69 ± 1.29	ND	0.20 ± 0.03	0.13 ± 0.08	0.66 ± 0.05	ND	25.51 ± 1.76	7.42 ± 0.58	0.03 ± 0.03	0.69 ± 0.20	0.04 ± 0.02	1.61 ± 0.28	1.30 ± 0.09	17.82 ± 0.62	3.20 ± 0.30	0.42 ± 0.06
PI	7.93 ± 1.35	1.74 ± 0.90	53.53 ± 0.99	1.71 ± 0.72	ND	ND	ND	ND	3.62 ± 0.77	ND	0.38 ± 0.22	0.21 ± 0.21	ND	ND	ND	8.73 ± 1.53	1.08 ± 0.64	16.85 ± 1.76	4.22 ± 0.59	ND
others	12.26 ± 0.12	0.90 ± 0.18	36.35 ± 1.39	3.70 ± 0.47	1.41 ± 0.66	ND	0.05 ± 0.05	0.07 ± 0.07	8.85 ± 3.46	ND	5.85 ± 1.32	1.57 ± 0.29	0.43 ± 0.34	0.28 ± 0.20	0.17 ± 0.17	4.19 ± 1.21	0.37 ± 0.22	3.75 ± 1.21	19.46 ± 1.50	0.32 ± 0.18

*Each value is represented as mol%. SE is based on four independent experiments. ND, not detected (<0.01 mol%).*

**Table 4:** Fatty acid composition of membrane lipids from *E*. *gracilis* cultured under Dark conditions.

	14:0	15:0	16:0	16:1	16:2	16:3	16:4	17:3	18:0	18:1(9)	18:1(11)	18:2	18:3 (*n*-6)	18:3 (*n*-3)	18:4	20:2	20:3 (*n*-6)	20:4	20:5	22:2
MGDG	1.66 ± 0.92	0.49 ± 0.52	3.66 ± 2.13	7.37 ± 4.86	19.23 ± 12.38	0.08 ± 0.00	2.70 ± 1.79	19.01 ± 9.23	0.69 ± 0.37	1.77 ± 2.93	4.66 ± 3.84	13.96 ± 8.88	0.06 ± 0.00	21.67 ± 10.45	ND	0.07 ± 0.00	0.24 ± 0.21	2.09 ± 1.43	0.59 ± 0.43	ND
DGDG	1.57 ± 0.12	ND	7.12 ± 0.27	4.77 ± 0.45	14.63 ± 0.51	ND	0.34 ± 0.17	25.24 ± 0.93	0.84 ± 0.13	ND	7.52 ± 0.80	11.89 ± 1.10	ND	25.28 ± 1.83	ND	0.07 ± 0.07	ND	0.67 ± 0.14	0.07 ± 0.07	ND
SQDG	12.29 ± 1.20	ND	59.78 ± 3.07	1.97 ± 1.00	4.15 ± 0.38	ND	ND	1.68 ± 0.92	4.50 ± 0.28	ND	5.80 ± 1.62	4.76 ± 0.78	ND	5.06 ± 0.08	ND	ND	ND	ND	ND	ND
PG	9.78 ± 2.02	ND	13.14 ± 2.10	10.06 ± 2.60	1.34 ± 0.71	ND	ND	ND	4.97 ± 1.21	ND	ND	17.00 ± 17.00	34.67 ± 17.33	ND	ND	ND	3.79 ± 0.91	3.57 ± 0.40	ND	1.67 ± 1.67
PC	12.69 ± 0.91	0.39 ± 0.09	16.54 ± 0.40	1.95 ± 0.09	0.07 ± 0.02	0.03 ± 0.03	0.09 ± 0.00	0.03 ± 0.03	0.81 ± 0.11	ND	5.25 ± 0.36	2.85 ± 0.20	0.53 ± 0.09	0.84 ± 0.09	0.32 ± 0.01	6.74 ± 1.03	12.95 ± 1.58	29.72 ± 1.09	6.24 ± 0.27	1.95 ± 0.12
PE	8.48 ± 1.52	0.44 ± 0.07	28.12 ± 0.92	10.82 ± 1.08	0.31 ± 0.03	ND	0.12 ± 0.01	0.02 ± 0.02	0.71 ± 0.16	ND	16.80 ± 2.11	4.93 ± 0.11	0.10 ± 0.10	0.30 ± 0.10	0.07 ± 0.00	2.17 ± 0.70	1.85 ± 0.11	22.43 ± 0.77	2.14 ± 0.19	0.20 ± 0.00
PI	11.96 ± 1.18	0.63 ± 0.63	58.11 ± 1.61	ND	ND	ND	ND	ND	6.14 ± 0.42	ND	ND	ND	ND	ND	ND	4.98 ± 0.17	0.42 ± 0.42	16.31 ± 0.39	1.44 ± 0.72	ND
Others	15.75 ± 1.19	0.42 ± 0.42	25.30 ± 1.40	2.01 ± 0.70	0.34 ± 0.20	ND	0.16 ± 0.16	ND	6.71 ± 0.31	ND	2.65 ± 1.07	0.90 ± 0.09	0.16 ± 0.16	1.05 ± 0.65	ND	5.85 ± 1.51	1.86 ± 0.61	10.16 ± 1.11	26.24 ± 1.77	0.43 ± 0.21

*Each value is represented as mol%. SE is based on three independent experiments. ND, not detected (<0.01 mol%).*

We also analyzed the abundancy of triacylglycerol (TAG) (**Table 1**), which is another storage lipid. The amount of TAG per cell increased when Glc was added to the medium, and TAG was highest in the cells grown under Dark conditions. This TAG had short chain saturated fatty acids, such as lauric acid 12:0, 14:0, and 16:0 as the major components (**Table 5**). On the other hand, the cells grown under CM or CM+Glc conditions had fewer short chain saturated fatty acids and more long chain fatty acids (20:4).

**Table 5:** Fatty acid composition of TAG from *E*. *gracilis* cultured under CM, CM+Glc and Dark conditions.

	12:0	13:0	14:0	15:0	16:0	16:1	16:2	16:4	17:3	18:0	18:1(11)	18:2	18:3 (n-6)	18:3 (n-3)	20:2	20:3 (n-6)	20:4	20:5	22:2
CM	5.30 ± 0.26	4.93 ± 0.99	18.52 ± 0.56	7.17 ± 0.17	27.43 ± 3.78	0.90 ± 0.90	0.43 ± 0.43	0.41 ± 0.41	ND	6.86 ± 2.14	7.03 ± 1.05	0.52 ± 0.27	ND	ND	1.86 ± 0.98	0.86 ± 0.44	9.39 ± 1.60	6.17 ± 1.53	2.2 ± 0.19
CM+Glc	4.81 ± 0.59	5.99 ± 0.68	21.77 ± 2.14	4.24 ± 1.07	14.09 ± 1.93	2.11 ± 0.59	2.38 ± 0.58	3.08 ± 1.73	1.04 ± 0.64	2.38 ± 0.25	4.83 ± 0.57	2.48 ± 0.69	ND	2.63 ± 1.46	4.56 ± 0.50	2.53 ± 0.59	14.53 ± 1.72	5.35 ± 0.56	1.2 ± 0.46
Dark	15.14 ± 1.50	4.07 ± 0.65	39.15 ± 2.65	1.54 ± 0.79	18.68 ± 0.70	5.67 ± 1.01	ND	ND	ND	2.13 ± 0.74	5.81 ± 2.79	1.33 ± 0.51	0.07 ± 0.07	ND	1.60 ± 0.33	0.89 ± 0.57	3.74 ± 1.61	0.19 ± 0.19	ND

*Each value is represented as mol%. SE is based on three independent experiments. ND, not detected (<0.01 mol%).*

## Discussion

### *E*. *gracilis* Uses Plant-Type Pathways for Galactolipid Synthesis

Euglenoids are believed to acquire chloroplasts through secondary endosymbiosis. We found that, under illumination, *E*. *gracilis* cells accumulated thylakoid glycolipids, especially two galactolipids, MGDG and DGDG. These galactolipids can be found in all oxygenic phototrophs and are known to be synthesized by two pathways, the plant-type pathway and cyanobacteria-type pathway (Awai, 2016). Some algae, such as primitive red algae and glaucophytes, are known to have the plant-type enzyme for MGDG synthesis and the cyanobacteria-type enzyme for DGDG synthesis (Awai et al., 2007; Sakurai et al., 2007; Hori et al., 2016; Maida and Awai, 2016; Sato and Awai, 2016). We analyzed whether the chloroplast glycolipids in *E*. *gracilis* are synthesized by the plant-type enzymes using EST data (Yoshida et al., 2016). As expected, the genes encoding plant-type enzymes for galactolipid synthesis were found (**Table 6**), as green algae utilize plant-type enzymes for both MGDG and DGDG synthesis. The SQDG synthetic pathway is basically conserved from cyanobacteria to plants, and *E*. *gracilis* was found to use the same system. We also tried to analyze the synthetic pathways for phospholipids determined by our analysis, but it was too complicated to be analyzed since euglenoids seem to have pathways from both the host cell and engulfed cell of the secondary symbiosis. Genomic sequencing analysis will be required to solve whole lipid synthetic pathway in *E*. *gracilis*.

**Table 6:** List of genes encoding homologs of thylakoid glycolipid synthases found in RNAseq data.

Genes	Function	Comp number in the RNAseq data
MGD1	Galactosyltransferase for MGDG synthesis (plant type)	comp33364_c0_seq1
		comp31220_c0_seq1
DGD1	Galactosyltransferase for DGDG synthesis (plant type)	comp33692_c1_seq1
		comp27690_c0_seq1
SQD2	Sulfoquinovosyltransferase for SQDG synthesis	comp34481_c0_seq2
		comp21910_c0_seq1

*The sequence data are available at the DDBJ Sequence Read Archive (DRA) with accession number SRP060591.*

### Possible Application for Bioengineering by Controlling Flow of Fatty Acid Metabolism

The production of biofuel for sustainable energy is highly anticipated as a next generation energy source for ecological and economic reasons (Chisti, 2007; Kraan, 2013). *E*. *gracilis* is a strong candidate for the production of such energy because of its ability to produce wax ester, which can exceed 40% of its dry weight (Inui et al., 1983). Wax ester in *E*. *gracilis* is synthesized from fatty acid and fatty alcohol mainly by WSD-type wax synthase (Tomiyama et al., 2017). This wax ester is suitable for bioenergy, because of their relatively short chained (C14) fatty acids and alcohols. Since the synthesis of membrane lipids and TAG also require fatty acids, it is important to know how the fatty acid flow and channel for synthesis of wax ester or glycerolipids. Our study described here provides details on how much glyceorolipids are synthesized in *E*. *gracilis* grown in photoautotrophic, photomixotrophic, and heterotrophic conditions. These data will inform the manipulation of fatty acid flow between glycerolipids and wax ester. We observed the accumulation of short chain fatty acid, especially 14:0 in TAG from cells grown under heterotrophic conditions. The major fatty acid of wax ester in *E*. *gracilis* is also 14:0, and TAG will be a target to enhance wax ester accumulation.

Recently, a photoenzyme which converts fatty acids to hydrocarbons was found in green algae (Sorigue et al., 2017). Using the amino acid sequence of this enzyme as a bait, we did a BLAST search of an Euglena EST database and found its homologs belonging to the GMC oxidoreductase super family (comp29747_c0_seq1: Expect = 6e-80 and comp30342_c0_seq1: Expect = 5e-40). However, enzymes of this family more closely resemble bacterial choline dehydrogenase, not the hydrocarbon synthase of green algae, implying that *E*. *gracilis* do not possess such enzymes for alkane synthesis. *E*. *gracilis* has the capacity to accumulate wax ester, and it is likely that it can be switch to synthesize alkene. Recently, a method for introduction of transgenes has been established in *E*. *gracilis* (Ogawa et al., 2015). It will be interesting to see if we can introduce this photoenzyme to *E*. *gracilis* with regulated fatty acid flow for another type of biofuel production in *E*. *gracilis*.

## Author Contributions

SS, TI, and KA conceived the research. SS and S-iA conducted the experiments. SS, S-iA, TI, and KA performed the data analysis and wrote the manuscript.

## Conflict of Interest Statement

The authors declare that the research was conducted in the absence of any commercial or financial relationships that could be construed as a potential conflict of interest. The reviewer WR and handling Editor declared their shared affiliation.

## Supplementary Material

FIGURE S1 SDS-PAGE of *E*. *gracilis* proteins. Proteins were extracted from cells grown under photoautotrophic, photomixotrophic and heterotrophic conditions and subjected to SDS-PAGE followed by CBB staining. Lane 1, CM conditions; lane 2, CM+Glc conditions; lane 3, Dark conditions. M: molecular weight marker (Nacalai Tesque, Inc.).
