# Supplementary figures and images for: Alterations of Membrane Lipid Content Correlated With Chloroplast and Mitochondria Development in Euglena gracilis
Source: Front Plant Sci. 2018 Mar 27;9:370. doi: 10.3389/fpls.2018.00370 (PMC5881160; doi:10.3389/fpls.2018.00370)

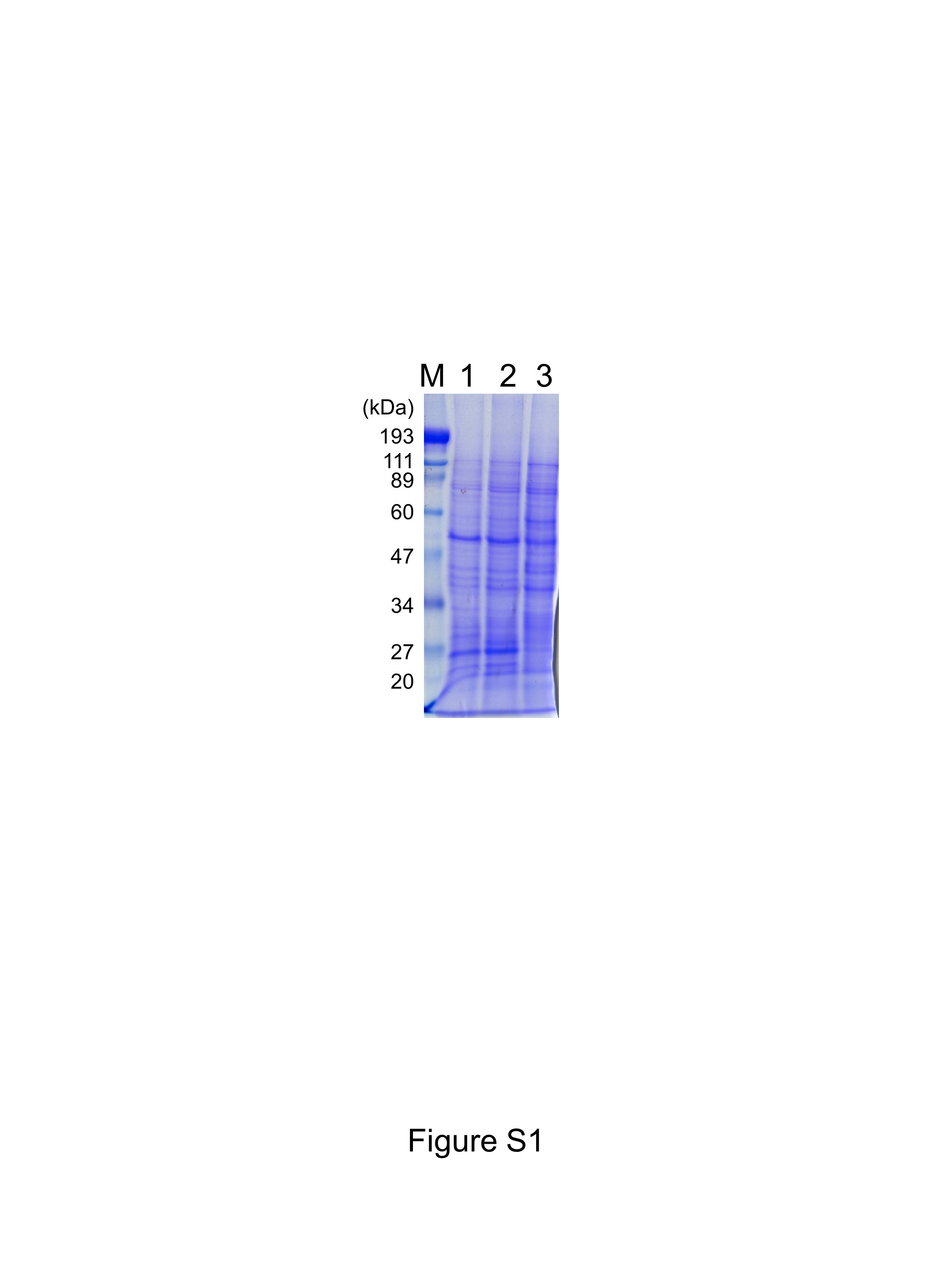

Supplement: FIGURE S1 — SDS-PAGE of E. gracilis proteins. Proteins were extracted from cells grown under photoautotrophic, photomixotrophic and heterotrophic conditions and subjected to SDS-PAGE followed by CBB staining. Lane 1, CM conditions; lane 2, CM+Glc conditions; lane 3, Dark conditions. M: molecular weight marker (Nacalai Tesque, Inc.). [file Image_1.JPEG]
